# Visible Light-Driven Photocatalytic Activity of Oleic Acid-Coated TiO_2_ Nanoparticles Synthesized from Absolute Ethanol Solution

**DOI:** 10.1186/s11671-015-1133-7

**Published:** 2015-10-23

**Authors:** Huihui Li, Bin Liu, Shu Yin, Tsugio Sato, Yuhua Wang

**Affiliations:** Key Laboratory for Magnetism Magnetic Materials of the Ministry of Education, Lanzhou University, 222 South Tianshui Road, Lanzhou, 730000 People’s Republic of China; Institute of Multidisciplinary Research for Advanced Materials, Tohoku University, 2-1-1 Katahira, Aoba-ku, Sendai, 980-8577 Japan

**Keywords:** TiO_2_, Visible light, Photocatalysis, Surface modification, Oleic acid

## Abstract

The one-step synthesis of oleic acid-coated TiO_2_ nanoparticles with visible light-driven photocatalytic activity was reported by this manuscript, using oleic acid-ethanol as crucial starting materials. The photocatalytic degradation of nitrogen monoxide (deNO_x_) in the gas phase was investigated in a continuous reactor using a series of TiO_2_ semiconductors, prepared from oleic acid- or acetic acid-ethanol solution. The surface modification on TiO_2_ by organic fatty acid, oleic acid, could reinvest TiO_2_ photocatalyst with the excellent visible light response. The deNO_x_ ability is almost as high as 30 % destruction in the visible light region (*λ* > 510 nm) which is similar to the nitrogen-doped TiO_2_. Meanwhile, acetic acid, a monobasic acid, has a weaker ability on visible light modification of TiO_2_.

**Electronic supplementary material:**

The online version of this article (doi:10.1186/s11671-015-1133-7) contains supplementary material, which is available to authorized users.

## Background

Visible light response modification has been one of the most important issues for photocatalysts, since TiO_2_ has large band gap energy of ca. 3.2 eV, which is active under irradiation of only UV light. Efforts to solve this problem have typically focused on many different modification strategies, although they often proceeded individually. One is focused on extending the range of excitation energies into the visible region by doping with elements to produce an excellent visible light responsive photocatalysts, such as N-doped TiO_2_, Cr-doped SrTiO_3_, and Ta- and N-codoped TiO_2_ [1–5]. Another one, the surface modification of a catalyst by dye sensitization or colorless organics, is also a good possibility to develop a method of designing a visible light responsive photocatalytic system. Dye-sensitized photocatalysis begins with the light absorption of dye and a subsequent electron transfer from the excited dye to the conduction band of the photocatalyst [6]. Various colorless organics acids, including ascorbic acid, salicylic acid, 5-sulfosalicylic acid, and so on have been used for the surface modification of TiO_2_. Attributed to the amount of surface hydroxyl groups introduced by these organic acids modification, the UV-vis wavelength response range of TiO_2_ photocatalyst was expanded and then modified TiO_2_ could be used as a visible light active photocatalyst [7–9]. In addition, such colorless organic acid surface-modified method was also designated as organic acid-coated method [7, 10]. Among various colorless organics surfactants, oleic acid is an excellent one because of its high affinity to the surface of superfine magnetite. The carboxyl groups from oleic acid could form ester-like linkages (C=O) or carboxylate linkages (C–O–O) with metal oxide, which played a positive role in the red-shift of the absorption edge of TiO_2_ or SrTiO_3_ nanoparticles [11, 12].

At the same time, the specific surface area, particle size, and crystal structure have a significant influence on photoefficiency. It is widely accepted that photocatalytic reactions mainly take place on the surfaces of photocatalysts [13]. The control of water generation speed, which plays an important role in the anhydrous solvothermal process, will result in samples with large specific surface area [14]. Acetic acid, which also possesses the carboxyl group, was used as one crucial starting material in such water-controlled release progress. However, the influences of acetic acid on the resulting products, the visible light activity mechanism, as well as the contribution of the work to the fields of visible light-driven photocatalysis were not reported or elaborated in detail. Oleic acid, an excellent surface modification agent, was expected to possess higher positive effect than acetic acid in such synthesis. The different effects of acetic acid and oleic acid on the carboxyl group-coated TiO_2_ particles prepared by solvothermal methods without water addition were studied.

Therefore, we expand on these aspects more explicitly in the current paper. There are two significant aspects of the work described in this paper. First, the synthesis of TiO_2_ particles in oleic acid-ethanol has been found to be successful. Hence, the absolute ethanol solution synthesis of TiO_2_ with nanosize should be an efficient progress that may inspire less aggregate material solvothermal synthesis. Second, photocatalysts with visible light activity has been intensively studied recently, but the use of oleic acid as a crucial starting material to design advanced photocatalysts with visible light activity in one-step reaction synthesis has been rarely reported. Hence, this work may be of interest to both photocatalysis scientists and those working in the area of visible light-driven photocatalyst design.

## Methods

The surface modification on TiO_2_ nanoparticles by oleic acid was conducted through a solvothermal method with controlling the speed of water generation. The typical preparation route was as follows: 1.70 g tetrabutyl titanate was added into a mixed solution consisting of 45-ml dehydrated ethanol and 5-ml oleic acid, under stirring. The resulting transparent solution was then transferred into a Teflon-lined autoclave, with an internal volume of 100 ml, followed by solvothermal reaction in an electric oven at 200 °C for 4 h. After the reaction, the products were centrifuged, washed four times alternately with water and ethanol, then, finally dried under vacuum at 60 °C. “TOL” is used to describe this sample. In order to investigate the effect of the amounts of Ti source, the amount of tetrabutyl titanate was changed to 0.24 g, and the product was designated as TOS. In addition, acetic acid was also used instead of oleic acid. “TAL” represents the sample prepared from acetic acid-ethanol solution with a large amount of Ti source, and “TAS” represents the sample from the acetic acid-ethanol solution using a little amount of Ti source. For comparison, 0.04 g of P25 TiO_2_ powders were dispersed in the same volume of oleic acid or acetic acid-ethanol solution followed by transferring into the same stainless steel autoclave with a Teflon tube. The autoclave was heated and kept at the same 200 °C for 4 h, then, finally washed and vacuum dried. TiO_2_ was also synthesized by adding 1 ml of water into the same starting materials. Nitrogen-doped TiO_2_ [1] and pure commercial TiO_2_ (P25, Degussa) were also used for comparison.

The X-ray diffraction (XRD) patterns of the catalysts were measured from 10° to 80° 2*θ* using a Shimadzu XD-D1 X-ray diffractometer and graphite-monochromic CuKα radiation. The catalyst morphology was observed by using an FEI Tecnai G2 F30 transmission electron microscope (TEM) with a Gatan imaging filter (GIF) system. The diffuse reflectance spectra (DRS) were determined using powder samples (Shimadzu UV-2450). The vibration spectra were characterized by Fourier transform infrared spectroscopy (FTIR) (NEXUS 670, Nicolet). The specific surface areas were determined by the amount of nitrogen adsorptions at 77 K (Quantachrome NOVA 4200e) using the Brunauer-Emmett-Teller (BET) method.

Photocatalytic activity during the oxidative destruction of NO was determined by measuring the concentration of NO gas at the outlet of the reactor (373 cm^3^) during photoirradiation under a constant flow of 1 ppm NO-50 vol.% air (balanced N_2_) mixed gas (200 cm^3^ min^−1^) [15]. Since illumination area is the key point in the continuous gas phase photocatalytic reaction, all powder photocatalysts were laid flat on the same glass holder with a certain area. Approximately 0.04 g of powder catalyst was placed into a 20 × 15 × 0.5 mm glass holder, which was kept in the bottom center of the reactor. The mixed gas with a flowing velocity of 200 cm^3^/min continuously passed through the reactor. A 450-W high-pressure mercury arc lamp was used as the light source, where the light wavelength was controlled by selecting various filters, i.e., Pyrex glass for cutting off the light of wavelength <290 nm, Kenko L41 Super Pro (W) filter <400 nm, and Fuji triacetyl cellulose filter <510 nm [15]. According to a Japanese Industrial Standard (JIS), the deNO_x_ characterization was carried out around room temperature. At the outlet of the reactor, the concentration of NO was determined using a NO_x_ analyzer (Yanaco, ECL-88A) [16]. The characterization system used in the present research was similar to that of the Japanese Industrial Standard which was established at the beginning of 2004 [17]. It has been reported that in the present characterization system during the photocatalytic destruction, about 20 % of NO is directly reduced to N_2_, and the other 80 % is oxidized to NO_3_^−^ species [18]. For comparison, the photocatalytic activity of commercial titania powders (P25, Degussa) were also determined.

## Results and discussion

**Fig. 1:**
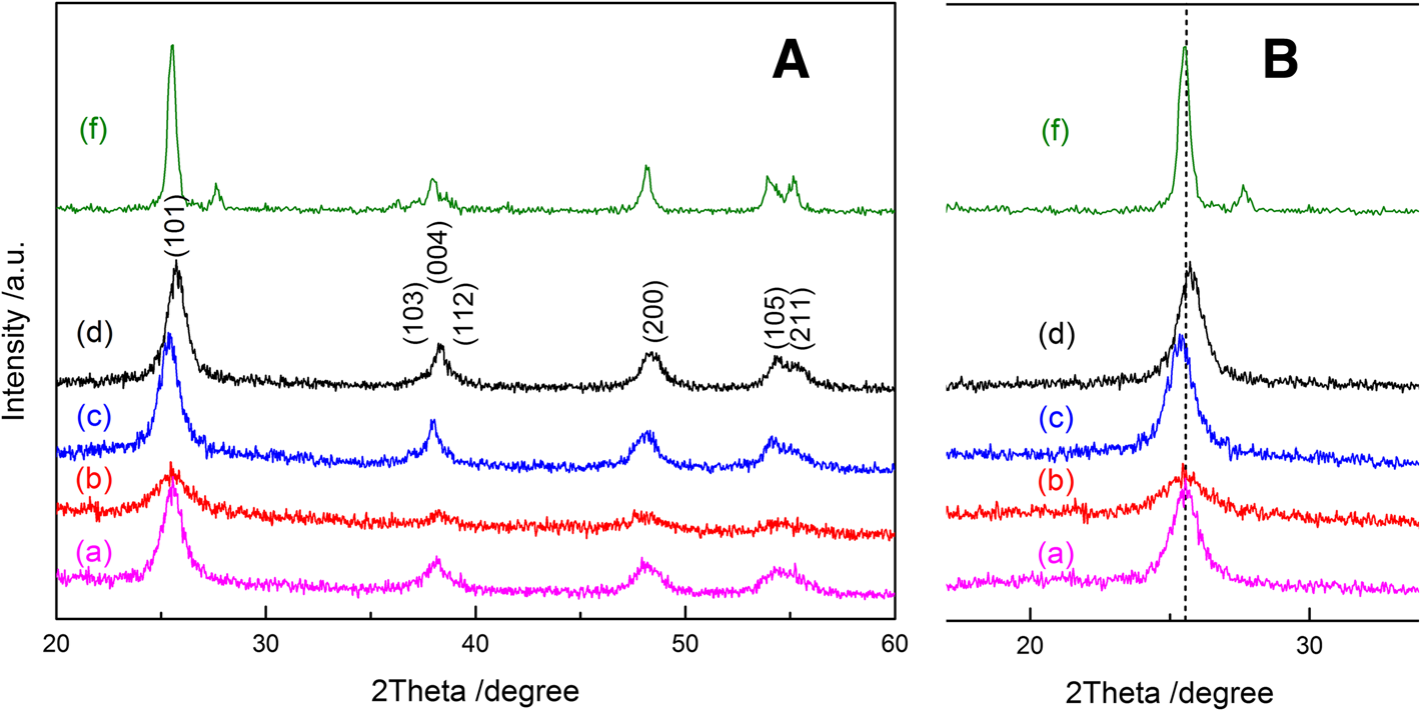
**a** XRD patterns of (*a*) TOL-, (*b*) TOS-, (*c*) TAS-, (*d*) TAL-TiO_2_, and (*f*) P25; **b** the magnified figure of **a** with the range from 20 to 30 2Theta degree

Figure 1 shows the XRD patterns of the powders prepared by the homogeneous precipitation-solvothermal treatment in (C_4_H_9_O)_4_Ti-oleic acid-ethanol solution or (C_4_H_9_O)_4_Ti-acetic acid-ethanol solution at 200 °C for 4 h. It can be seen that the sample consisted of single phase of anatase (JCPDS No. 021-1272) without remaining precursor. Besides anatase phase, P25 showed a characteristic peak at 27.4° attributed to rutile phase (JCPDS 065-0190), since P25 is a mixture of anatase and rutile. Certain Bragg peaks ({101} crystal facets) of as-prepared pure TiO_2_ samples showed a slight shift due to the formation of defects in the crystalline lattice of anatase. Those peaks observed for the samples from oleic acid-ethanol solution with a large amount of Ti source were broad due to the anisotropic growth of the nanoplates along the *c*-axis of the anatase lattice [19]. This indicates that the addition of acetic acid and oleic acid played different roles in the crystal structure formation.

**Fig. 2:**
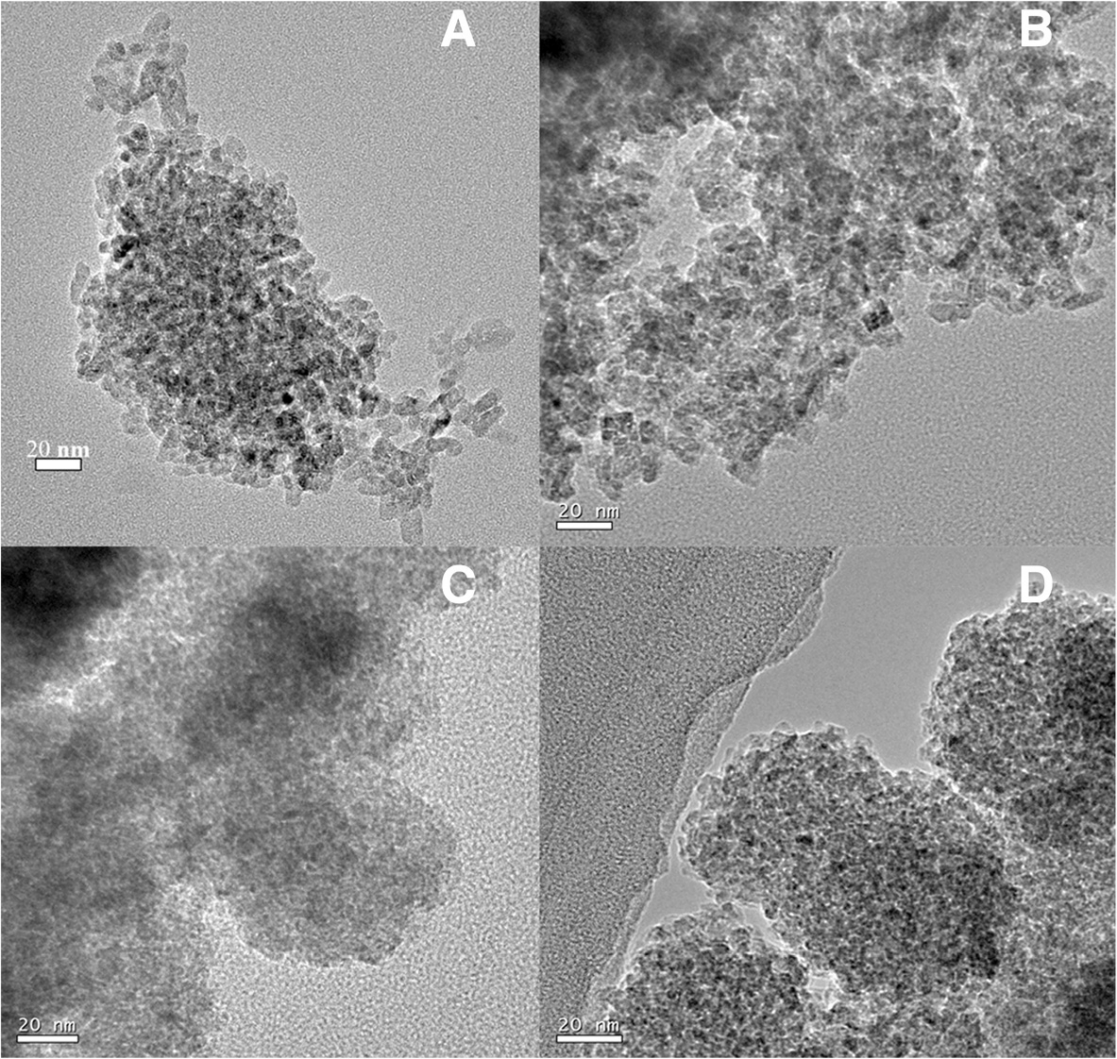
TEM images of the **a** TAL-, **b** TAS-, **c** TOS-, and **d** TOL-TiO_2_ samples

Figure 2 shows the TEM photographs of the powders prepared in (C_4_H_9_O)_4_Ti-oleic acid-ethanol solution via solvothermal treatment at 200 °C. It can be seen from Fig. 2a, b, d that the TiO_2_ crystallite sizes were around 15 nm, while those shown in Fig. 2c was smaller than 10 nm. The mixed ethanol solvent had a smaller dielectric constant and could contribute to the decrease in solubility of titania during the crystallization process. As a result, the smaller particles were prepared. Meanwhile, owing to the chemical adsorption of oleic acid on the surface of TiO_2_, the crystal growth during the solvothermal treatment at 200 °C was greatly suppressed. The crystals prepared from the oleic acid-ethanol solution showed a smaller particle size (Fig. 2c, d) than those synthesized via the addition of acetic acid to ethanol solution. In addition, the effect of the adding amount of Ti source on particle size was limited, i.e., there was no significant distinction between two TiO_2_ nanoparticles via a large amount of Ti source or small one. Table 1 shows the BET specific surface areas (S.S.A.) of the powders prepared in various ethanol solutions with oleic acid or acetic acid addition. According to the results, the as-prepared TiO_2_ samples from absolute ethanol solution were nanoparticles with large specific surface areas. In addition, the powders obtained in oleic acid-ethanol solution with a large amount of Ti source possessed similar specific surface areas to those obtained in acetic acid addition one. TOS-TiO_2_ nanoparticles showed a larger specific surface area of 183.1 m^2^/g. It might be attributed to the inhibitory effect of oleic acid on crystal growth. The effect was more efficient when the small amount of Ti source was added.

**Table 1:** Specific surface areas of the TiO_2_ samples

Sample	TAL	TAS	TOL	TOS	P25
S.S.A. (m^2^/g)	162.8	167.4	162.3	183.1	47.0

**Fig. 3:**
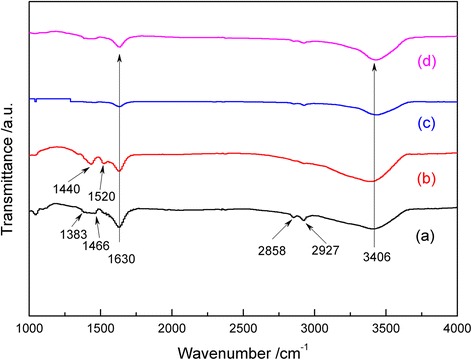
FTIR spectra of the as-prepared TiO_2_ samples (*a*, *b*) synthesized in the ethanol-oleic acid (*a*) or acetic acid (*b*) solution and the as-treated P25 TiO_2_ (*c*, *d*) from the same ethanol-oleic acid (*c*) or acetic acid (*d*)

The FTIR spectra of the samples synthesized from an oleic acid-ethanol solution or an acetic acid-ethanol solution after solvothermal reaction are shown in Fig. 3. For comparison, the FTIR spectra of the processed P25 TiO_2_ powder samples are also shown in Fig. 3c, d. These P25 TiO_2_ powder were dispersed in oleic acid or acetic acid-ethanol and heated in a sealed stainless steel autoclave at 200 °C for 4 h. FTIR analysis suggests the peaks around 1500 cm^−1^ corresponding to a carbon ingredient even P25 TiO_2_ powders washed and dried after being dispersed and heated in organic acid-ethanol solution. During treatment of all the TiO_2_ samples by the solvothermal reaction, their surfaces were readily covered with hydroxyl groups in an aqueous environment [12, 20] which was formed by slowly releasing water from ethanol [14]. Thus, the characteristic bands of hydroxyl groups, 1630 and 3406 cm^−1^, appeared in the FTIR spectra. Compared with other TiO_2_ powders, two new bands at 2858 and 2927 cm^−1^ appeared in FTIR spectrum of TiO_2_ nanoparticles synthesized by limited oleic acid (TOL, Fig. 3a). These two bands were attributed to the CH_2_ asymmetric and CH_2_ symmetric stretch, respectively [21]. It is worth noting that the intense peak at 1708 cm^−1^ assigned to the stretching vibration of C=O in oleic acid [22] was absent in the spectrum of TiO_2_ particles from oleic acid-ethanol solution. Instead, four new bands at 1383 and 1466 cm^−1^ appeared in the FTIR spectrum of TOL TiO_2_ while 1440 and 1520 cm^−1^ appeared in that of TAL TiO_2_, respectively. The band at 1383 and 1440 cm^−1^ could be assigned as the symmetric stretch of COO^−^, and the vibration bands at 1466 and 1520 cm^−1^ could be assigned as the asymmetric stretch of COO^−^ [12, 20]. This suggests that the oleic acid was chemisorbed as carboxylate on the surface of TiO_2_. Based upon previous studies [23], the interaction between the carboxylate and metal atom is categorized into four types: monodentate, bridging bidentate, chelating bidentate, and ionic interaction. The wave number separation (Δ) between asymmetric and symmetric stretch IR bands can be used to analyze the type of interaction between the carboxyl and metal atom. The largest separation (200–320 cm^−1^) corresponds to the monodentate interaction, and the smallest one (<110 cm^−1^) is for the chelating bidentate. The medium range (140–190) is for the bridging bidentate. In the present work, Δ could be found as 80 and 83 cm^−1^. This indicates that there was one kind of interaction of carboxyl and metal on the surface of the sample, chelating bidentate (Δ = 80 or 83 cm^−1^).

**Fig. 4:**
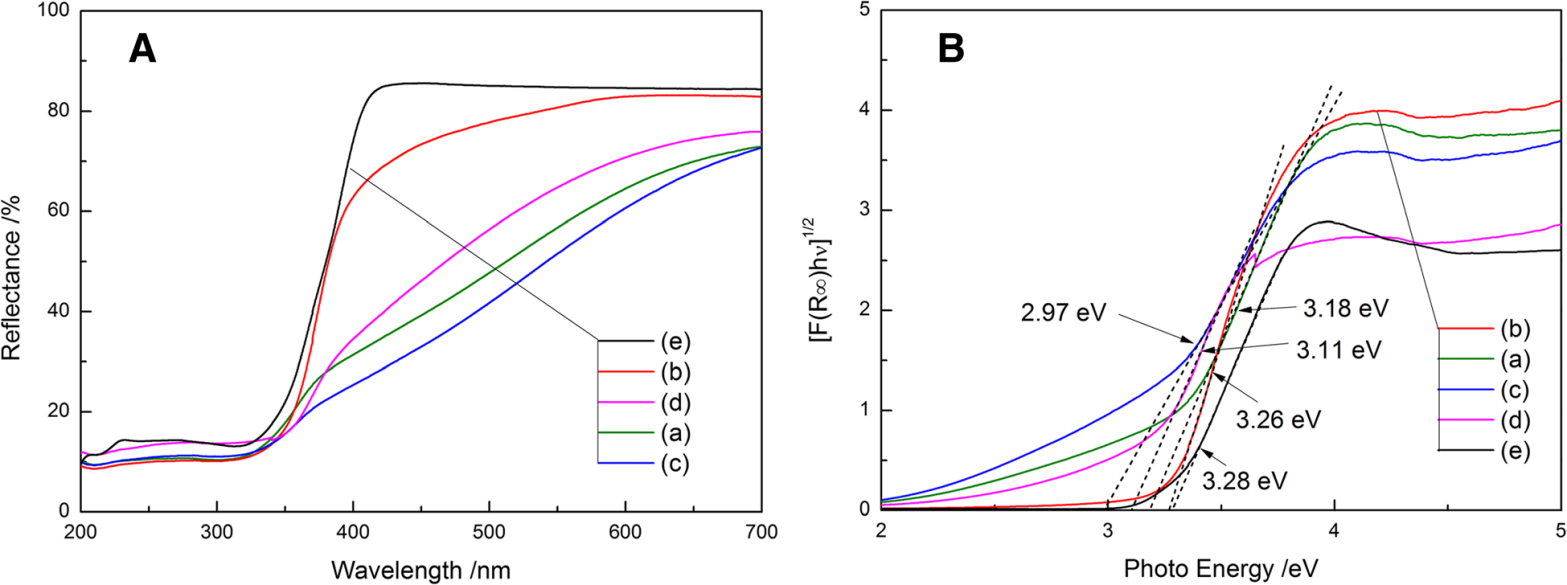
**a** Diffuse reflectance spectra and **b** Kubelka-Munk plots of various TiO_2_ powders. (*a*) TAL, (*b*) TAS, (*c*) TOS, (*d*) TOL, and (*e*) P25

Figure 4 shows the diffuse reflectance spectra of the TiO_2_ powders prepared by solvothermal treatment in (C_4_H_9_O)_4_Ti-oleic acid-ethanol solution or (C_4_H_9_O)_4_Ti-acetic acid-ethanol solution. The band gap energies were calculated using the Kubelka-Munk method based on the diffuse reflectance spectra, where *F*(R) = (1−R)^2^/2R [19]. It can be seen that all samples showed absorption edges around 400 nm due to the band gap absorption of anatase TiO_2_. Except TAS TiO_2_ with the band gap energy of 3.26 eV, the as-prepared anatase TiO_2_ nanoparticles possessed lower band gap energies, 2.97, 3.11, and 3.18 eV respectively, than pure anatase TiO_2_ which is ca. 3.2 eV. As expected, P25 showed the poor response in the visible light region due to its large band gap energy of ca. 3.28 eV. It is worthy noting that neither oleic acid nor acetic acid did not change the band structure of TiO_2_ while enhanced the visible light absorption by organic acid surface modification. The absorption of as-prepared TiO_2_ samples in the visible light range of 400–700 nm increased in the order:

TOS > TAL > TOL > TAS > P25

The visible light absorption of TiO_2_ particles prepared from organic acid-ethanol solution can be explained as follows: a dipole layer has been formed towards the inner TiO_2_ through the carboxyl group binding with the Ti^4+^. The carboxyl groups come from oleic or acetic acid, which is chemically adsorbed on the surface of TiO_2_ particles. This dipole layer induces an attracting potential for electrons instead of the TiO_2_ nanoparticles, which contributes to the absorption in the visible region. Similar phenomenon has also been reported by SrTiO_3_ bound by oleic acid and TiO_2_ bound by propionic acid and *n*-hexylamine on the surface showing a dipole layer formation [11, 12]. This dipole layer contributes to the reduction of the band gap of as-prepared anatase TiO_2_, leading to the red-shift of the absorption band edge.

**Fig. 5:**
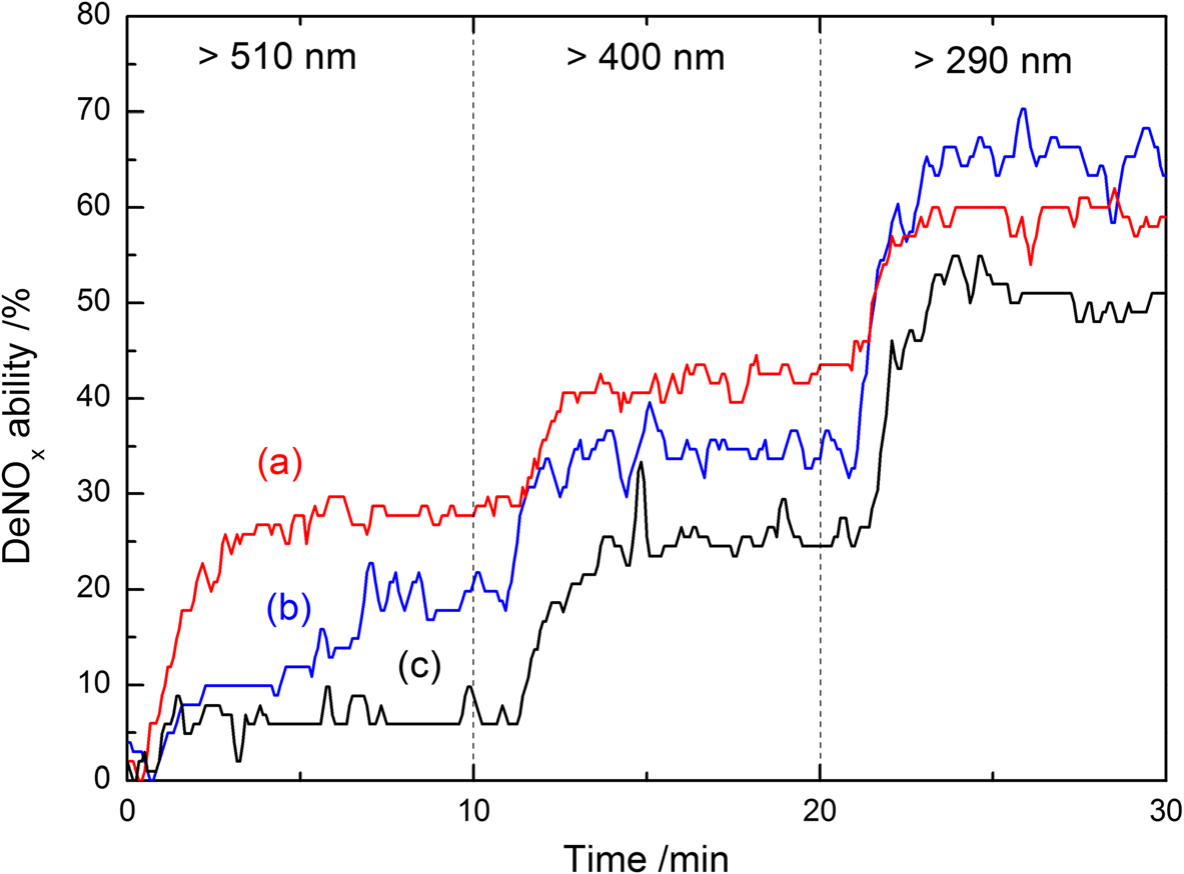
Photocatalytic activity for the oxidation of nitrogen monoxide of the prepared samples during various wavelengths light irradiation: (*a*) TOS-TiO_2_, (*b*) TAL-TiO_2_, and (*c*) commercial powder Degussa P25 TiO_2_

To investigate the photocatalytic activity of the prepared samples, the oxidative photo destruction of NO_x_ gas was performed as a model reaction. Figure 5 shows the photocatalytic deNO_x_ ability of the TiO_2_ nanoparticles prepared by the homogeneous precipitation-solvothermal treatment in (C_4_H_9_O)_4_Ti-oleic acid-ethanol solution or (C_4_H_9_O)_4_Ti-acetic acid-ethanol solution at 200 °C for 4 h. For comparison, P25 was used as a standard sample. According to Fig. 5, the organic acid-modified TiO_2_ showed higher photocatalytic deNO_x_ abilities than P25 TiO_2_ under not only UV light but also visible light irradiation. Oleic acid-coated titania (TOS) and acetic acid-coated titania (TAL) possessed visible light-induced activity under visible light irradiation with two different wavelengths (>510 or >400 nm). These results are consistent with the DRS spectra in that P25 TiO_2_ was able to absorb only UV light, while organic acid-coated TiO_2_ was able to absorb both UV and some degree of visible lights. It is notable that under irradiation in the visible light region (*λ* > 510 nm), the deNO_x_ ability of P25 is modest, i.e., showing photodegradation of NO_x_ less than 6 %. In contrast, the TiO_2_ nanoparticles from oleic acid (TOS) or acetic acid-ethanol solution (TAL) presented high photocatalytic activities. After 10 min irradiation, the NO_x_ degradation efficiency reached 28.6 % for TOS TiO_2_ and 19.8 % for TAL TiO_2_, respectively. The dipole layer caused by the chemical adsorption of oleic or acetic acid results in the visible light response of organic acid-coated titania photocatalyst, and that from oleic acid was more competent. However, TiO_2_ samples prepared via the same solvothermal reaction but adding a different amount of Ti source (TOL and TAS) exhibited nearly the same activities as P25, as shown in Additional file 1: Figure S2. It suggests that there are some differences in the relationship between organic acid surface modification and the concentration of starting materials. In the present work, it is a good idea to combine low concentration of Ti source with oleic acid while high one with acetic acid. The relationship between organic acid surface modification (including fatty acid and monobasic acid) and the concentration of starting materials will be investigated and discussed in more details in future. In addition, nitrogen-doped TiO_2_ (N-TiO_2_) with the same anatase phase and specific surface area of 250.0 m^2^/g (Additional file 1: Figure S3) was used for comparison. As seen in Additional file 1: Figure S4, N-TiO_2_ nanoparticles possessed high visible light response due to the narrowed band gap of titania caused by the valence band of N2p band locating above O2p band [24]. Surprisingly, TOS presented photocatalytic activity as high as that of N-TiO_2_ under visible light irradiation with the wavelength of longer than 510 nm (Additional file 1: Figure S5). It is well known that photocatalytic activity is strongly dependent on physical properties such as the crystal phase, particle size, specific surface area, morphology, and so forth. In this work, considering that all samples possessed the similar crystal phase and specific surface area, the excellent photocatalytic activity of TOS prepared from oleic acid-ethanol solution with adding a little amount of (C_4_H_9_O)_4_Ti is mainly due to its stronger absorption of visible light, which creates more photo-induced electrons and holes. This is facilitated by the formation of a dipole layer on the surface of TiO_2_ [11, 12]. The dipole layer contributes to the absorption of visible light; therefore, this layer might become the trap center of the exciton, which could enhance the exciton binding energy more significantly [25]. It agreed well with the results in our previous work, which suggested the fatty acid played an important role in the visible light activity enhancement by surface modification [26]. According to the result that acetic acid is inferior to oleic acid in surface modification, acetic acid gave way to oleic acid in the absolute ethanol solution to prepare photocatalyst with high visible light activity. In a previous work, the photocatalyst showed stable activity for the repeated deNO_x_ reaction for 120 min after the functional group of oleic acid chemically bonded on the surface [12].

Table 2 summarized the average light absorption degrees, photocatalytic deNO_x_ abilities, and apparent quantum efficiencies (QE_a_) of different TiO_2_ samples under irradiation of high-pressure mercury arc (>510 nm). QE_a_ was calculated according to Eq. (S1) (see Additional file 1) [2, 27]. It is clear that TOS nanoparticles possessed much higher *QE*_*a*_ under visible light irradiation with the wavelength of longer than 510 nm, due to the higher ability of visible light absorption. This result indicates that TOS photocatalyst can utilize visible light with long wavelength more effectively than other TiO_2_ samples.

**Table 2:** Physical properties and photocatalytic abilities for the destruction of nitrogen monoxide under visible light irradiation

TiO_2_ samples	Visible light region (*λ* > 510 nm)		
	A^a^ (%)	DeNO_x_ (%)	QE_a_ (%)
TAL	36.2	20.0	0.106
TAS	17.9	5.6	0.060
TOS	39.4	28.6	0.138
TOL	30.0	8.9	0.057
P25	15.5	5.8	0.072

^a^The average light absorption degrees of the samples

Finally, it is important to note that this organic acid-absolute ethanol solution synthesis applies to the situation that starting materials exhibit good solubility in such organic solvents only. For example, SrTiO_3_ crystals could not be prepared successfully from such ethanol solution by using SrCl_2_ as Sr source (as shown in Additional file 1: Figure S1).

## Conclusions

In this work, we successfully prepared oleic acid-coated anatase TiO_2_ nanoparticles with high visible light activity from absolute ethanol solution. The functional group (−COO^−^) of oleic acid chemically bonded on the surface generated new absorption in the visible region and induced visible light responsive photocatalytic activity. In addition, acetic acid loses out to oleic acid in respect of visible light modification, although the dipole layer was formed in acetic acid-coated TiO_2_.

## Additional files

Additional file 1:
**Supplementary information.** **Figure S1.** XRD patterns of as-prepared samples by adding Sr source (SrCl_2_) into starting material (a) oleic acid-ethanol and (b) acetic acid-ethanol solutions with a large amount of Ti source. **Figure S2.** DeNO_x_ abilities of different TiO_2_ samples. **Figure S3.** Crystalline morphology properties of nitrogen-doped TiO_2_ nanoparticles. **Figure S4.** DRS spectrum of nitrogen-doped TiO_2_ and P25 TiO_2_. **Figure S5.** DeNO_x_ abilities of nitrogen-doped and TOS-TiO_2_ samples.
