# Supplementary material for: Visible Light-Driven Photocatalytic Activity of Oleic Acid-Coated TiO2 Nanoparticles Synthesized from Absolute Ethanol Solution
Source: Nanoscale Res Lett. 2015 Oct 23;10:415. doi: 10.1186/s11671-015-1133-7 (PMC4615996; doi:10.1186/s11671-015-1133-7)
Supplement: Additional file 1: — Supplementary information. Figure S1. XRD patterns of as-prepared samples by adding Sr source (SrCl2) into starting material (a) oleic acid-ethanol and (b) acetic acid-ethanol solutions with a large amount of Ti source. Figure S2. DeNOx abilities of different TiO2 samples. Figure S3. Crystalline morphology properties of nitrogen-doped TiO2 nanoparticles. Figure S4. DRS spectrum of nitrogen-doped TiO2 and P25 TiO2. Figure S5. DeNOx abilities of nitrogen-doped and TOS-TiO2 samples. [file 11671_2015_1133_MOESM1_ESM.doc]

Visible light driven photocatalytic activity of oleic acid-coated TiO_2_ nanoparticles synthesized from absolute ethanol solution

*Huihui Li^a,*^, Bin Liu^a,*^, Shu Yin^b^, Tsugio Sato^b^, Yuhua Wang^a^*

^a^ Key Laboratory for Magnetism Magnetic Materials of the Ministry of Education, Lanzhou University, 222 south Tianshui Road, Lanzhou 730000, PR China

^b^ Institute of Multidisciplinary Research for Advanced Materials, Tohoku University, 2-1-1 katahira, Aoba-ku, Sendai 980-8577, Japan

* Corresponding author at: Key Laboratory for Magnetism Magnetic Materials of the Ministry of Education, Lanzhou University, 222 south Tianshui Road, Lanzhou 730000, PR China. Fax: +86-0931-8912554

E-mail addresses: [lihh@lzu.edu.cn](mailto:lihh@lzu.edu.cn) (H. Li), [liubin@lzu.edu.cn](mailto:binliu@lzu.edu.cn) (B. Liu)

1. Photocatalytic reaction supplementary information

The photocatalytic activity for nitrogen monoxide destruction was determined by measuring the concentration of NO gas at the outlet of the reactor (373 cm^3^) during the photoirradiation of constant flowed 1 ppm NO 50 vol.% air-mixed (balance N_2_) gas (200 cm^3^/min). The photocatalyst was placed in a hollow place of 20×15×0.5 mm on a glass holder/plate and set in the center of the reactor. A 450-W high-pressure mercury lamp was used as the light source, where the light wavelength was controlled by selecting various filters. The experimental apparatus and the wavelength distribution of the light irradiated from a 450-W high-pressure mercury lamp were reported in a previous paper in detail [S1].

The characterization system used in the present research was similar to that of the Japanese Industrial Standard which was established at the beginning of 2004[S2]. In this JIS standard, it is recommended that the photocatalytic activity of photocatalyst should be characterized by measuring the decrease in the concentration of NO at the outlet of a continuous reactor. One ppm of NO gas with a flow rate of 3.0 dm^3^/min is introduced to a reactor then irradiated by a lamp with light wavelength of 300-400nm.

The mechanism of photocatalytic deNOx had been researched carefully by M.Anpo. During the deNOx photocatalytic reaction, the nitrogen monoxide reacts with these reactive oxygen radicals, molecular oxygen, and very small amount of water in air to produce HNO_2_ or HNO_3_. It was confirmed that about 20% of nitrogen monoxide was decomposed to nitrogen and oxygen directly [S3].

*References:*

(S1) Yin, S.; Maeda, D.; Ishitsuka, M.; Wu, J.; Sato, T. Synthesis of HTaWO_6_/(Pt, TiO_2_) nanocomposite with high photocatalytic activities for hydrogen evolution and nitrogen monoxide destruction, Solid State Ionics, **2002,** 151, 377-383.

(S2) Japanese Industrial Standard (JIS R 1701-1:2004(J)), Test method for air purification performance of photocatalytic materials – Part 1: Removal of nitric oxide*,* Japanese Standards Association, Established on 2004-01-20.

(S3) Anpo, M. in *“Recent Development on Visible Light Response Type Photocatalyst”* (ISBN4-86043-009-03), NTS, Tokyo **2002,** p9.

2. Supplementary data information





Fig. S1 XRD patterns of as-prepared samples by adding Sr source (SrCl_2_) into starting material (a) oleic acid-ethanol and (b) acetic acid-ethanol solutions with a large amount of Ti source.





Fig. S2 DeNO_x_ abilities of different TiO_2_ samples.


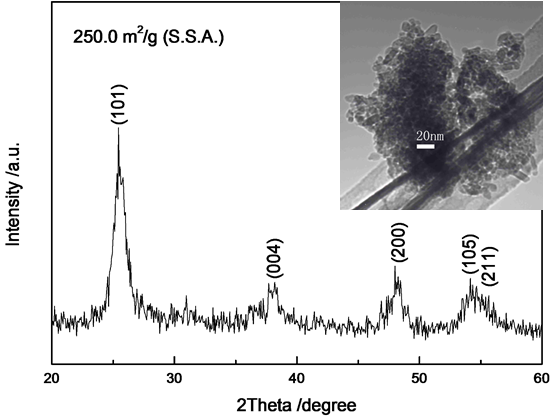


Fig. S3 Crystalline morphology properties of nitrogen-doped TiO_2_ nanoparticles.





Fig. S4 DRS spectrum of nitrogen-doped TiO_2_ and P25 TiO_2_.





Fig. S5 DeNO_x_ abilities of nitrogen-doped and TOS-TiO_2_ samples.

Light amount for photocatalysis.

Under mercury arc irradiation

$QE_{a,445nm}=\frac{F_{NO}\alpha_{\lambda}}{P_{1}SA_{\lambda}}\times100\%$ (S1)

*F_NO_* = (200×10^-3^ L÷60 s)×10^-6^÷22.4 L mol^-1^ = 1.488×10^-4^ μmol s^-1^ (NO, 1 ppm, 200 ml/min)

*P_1_* = 337 *μmol m^-2^ s^-1^* (Light intensity of mercury arc (>510 nm)) [S1]

$\alpha_{\lambda}$is deNO_x_ ability of photocatalyst under irradiation.

S = 3.2×10^-4^ m^2^

*A_λ_* is the absorption ability to mercury arc light with the wavelength of longer than 510 nm.

**References**

(S1) Yin, S.; Ihara, K.; Aita, Y.; Komatsu, M.; Sato, T. [Visible-light induced photocatalytic activity of TiO_2−_*_x_*A*_y_* (A = N, S) prepared by precipitation route](http://www.sciencedirect.com/science/article/pii/S101060300500393X). *J. Photochem. Photobiol. A* **2006**, 179, 105-114.
